# Genetic mutation of SLC6A20 (c.1072T > C) in a family with nephrolithiasis: A case report

**DOI:** 10.1515/med-2023-0648

**Published:** 2023-02-14

**Authors:** Menglei Jv, Jing Zheng, Anni Yang, Wei Xie, Weiping Zhu

**Affiliations:** Department of Nephrology, The Fifth Affiliated Hospital of Sun Yat-Sen University, Zhuhai, China; Department of Rheumatology, The Fifth Affiliated Hospital of Sun Yat-Sen University, Zhuhai, China; Department of Otolaryngology Head and Neck Surgery, The Fifth Affiliated Hospital of Sun Yat-Sen University, Zhuhai, China

**Keywords:** SLC6A20, nephrolithiasis, whole exome sequencing, gene variation

## Abstract

Nephrolithiasis is a highly prevalent disease worldwide that is associated with significant suffering, renal failure, and cost for the healthcare system. A patient with nephrolithiasis was found to have SLC6A20 variation. SLC6A20 gene in human is located on chromosome 3p21.3, which is a member of SLC6 family of membrane transporters and the product of this gene expression is transporter protein of sub-amino acid transporter system. The previous studies have reported that the mutation of SLC6A20 may cause hyperglycinuria or iminoglycinuria which may lead to nephrolithiasis. The object was to investigate the relationship between nephrolithiasis and SLC6A20 through pedigree genetic analysis. To explore whether the SLC6A20 mutation can cause hereditary nephrolithiasis, and provide evidence for further research. The urine and blood were collected from the patients for compositional analysis. DNA sequencing was applied to analyze the gene mutation. Labial gland and kidney biopsy were conducted for pathological analysis. As a result we reported a rare family case of nephrolithiasis accompanied by primary Sjogren’s syndrome and investigated it by examining the family members with whole exome gene sequencing technology and detecting 20 different amino acids and 132 kinds of organic acids in the urine with gas chromatography-mass spectrometry. We discovered that the proband and her mother had hyperglycinuria and the proband (Ⅱ_2_), her sister (Ⅱ_3_), and mother (Ⅰ_1_) were found to carry the SLC6A20 gene exon NM_020208.3 sequence c.1072T > C heterozygous mutation, and the other family members (Ⅰ_2_, Ⅱ_1_, Ⅱ_4_, Ⅲ_1_, Ⅲ_2_) did not carry the genetic mutation. As a conclusion, the heterozygous mutation of SLC6A20 (c.1072T > C) might be contributed to hyperglycinuria and the formation of nephrolithiasis.

## 1 Introduction

Nephrolithiasis is a highly prevalent disease worldwide that is associated with significant suffering, renal failure, and cost for the healthcare system. In recent years, the morbidity rate of nephrolithiasis has significantly increased. The prevalence of kidney stones ranges from 3 to 14% worldwide and it is still increasing dramatically [1,2]. The total prevalence of kidney stones in China is reported to be 7.54% in recent studies [3,4]. As a common urinary system disease, nephrolithiasis is caused by abnormal accumulation of crystal substances in the kidney. The incidence in male is more than that in female, and most of them occur in young adults. Calcium oxalate and calcium phosphate calculi are most common calcic stone, which account for more than 80% of nephrolithiasis. Uric acid stones account for 5–10% of all kidney stones [5]. Less common stone types include cystine, magnesium ammonium phosphate, and infectious stones [6].

The etiology of nephrolithiasis is considered to be related with heredity, age, sex, hardness of water, and environmental factors [7–10]. Numerous studies have indicated that the immediate family members of nephrolithiasis patients are highly susceptible to develop nephrolithiasis [11]. Genome-wide association studies have identified variants at the CLDN14, CaSR, TRPV5, ALPL, SLC34A1, AGXT, ATP6V1B1, CLDN16, CLDN19, GRHPR, SLC3A1, SLC9A3R1, SLC12A1, SLC26A1, and CYP24A1 as risk factors for nephrolithiasis and contributors to familial aggregation [12–14]. Furthermore, several studies have discovered that polymorphisms in PDE1A (rs182089527), HIPK2 (rs2058265), Alpha-2-MRAP, CALCR (rs1801197), SLC13A2 (rs11567842), and VDR (rs1544410) are associated with an increased risk of nephrolithiasis [15–20]. The genetic diagnosis of these causative mutations may provide specific guidance for stone prevention and management. SLC6A20 is a sodium chloride-dependent transporter in the sub-amino acid transporter system [21] and has been associated with some forms of autosomal recessive sub-amino aciduria [22]. More than 100 variation locations in SLC6A20 have been reported in ClinVar, which may also cause hyperglycinuria or iminoglycinuria. The free glycine might increase the urinary oxalate concentration and promote oxalate calculus formation [23,24].

In this study, we report a rare case found in a family with nephrolithiasis and primary Sjogren’s syndrome (pSS), which have a tendency for aggregation and are closely associated with the heterozygous variation c.1072T > C in the SLC6A20 gene.

## 2 Case presentation

A 34-year-old woman with no previous medical history had dry mouth, foamy urine, and increased nocturnal symptoms for 1 year. She came from a family of non-close relatives marriage and was found to have bilateral nephrolithiasis in a physical examination. Physical examination revealed no significant abnormalities. A 32-year-old woman, the patient’s younger sister also had dry mouth and increased nocturia symptoms for 2 years. She also has no previous medical history. There is no other family members who had pSS. Their father has a history of hypertension and type 2 diabetes but no nephrolithiasis. Their mother had a history of nephrolithiasis and had received extracorporeal shock wave lithotripsy. The children of both sisters were in good health and did not have nephrolithiasis or pSS.

## 3 Methods

### 3.1 Sample collection

With the informed consent of the patients and their relatives and the approval of the hospital ethics committee, blood and urine samples of them were collected.

### 3.2 Pathological analysis

According to the guideline [25], the labial gland tissues of the sisters were obtained by biopsy and stained by histochemistry; the kidney tissues were obtained by biopsy of the kidney and analyzed by immunofluorescence, histochemistry, and transmission electron microscopy.

### 3.3 Analysis of amino acids and organic acids in urine

Filter paper (5 cm × 5 cm) was used to collect morning urine or 24 h urine samples, which were dried and then put into special filter paper drying bag for inspection. The dried filter paper samples were centrifuged and eluted with 2 mL deionized double distilled water several times. After extraction and derivatization of the re-soluble liquid, 1 mL of the sample was taken. Samples were analyzed by gas chromatography-mass spectrometry (Shimadzu QP2010, JAPAN). The mass spectrometric samples were introduced from the GC inlet and separated by a four bar mass analyzer according to the mass load ratio. The signal is processed by computer and the corresponding peak area and characteristic ion pair are obtained. Compared with the standard spectrum library, the characteristic ion pair is used to determine the quality and the peak area, and then combined with the clinical data to analyze.

### 3.4 DNA sequencing

About 5 mL venous blood was taken from each affected member of the family and the genomic DNA was extracted according to the manufacturer’s standard procedure of the MagPure Buffy Coat DNA Midi KF Kit (Magen, Guangzhou, China). Then the genomic DNA was fragmented by Segmentase (BGI, China). The DNA fragments were amplified by ligation-mediated polymerase chain reaction and purified to form a library. Then, the library was enriched. All amplified libraries were subsequently sent to Beijing Genomics Institute (BGI) for circularization and sequencing on the MGIseq-2000 platform with a sequencing strategy of Paired-end 100. After sequencing, the data were automatically demultiplexed by index. Then evaluated the sequencing quality of the original data to remove the low quality and contaminated reads. Burrows Wheeler Aligner (BWA) software (burrows Wheeler aligner) and hg19/HG20 were used for sequence alignment, and the effect of sequence capture was evaluated. Genome Analysis Toolkit (GATK) software was used for single nucleus variant (SNV) and Indel (insertion and deletion) queries to generate the results of base polymorphism in the target region. Then the database was compared and the suspicious mutations were annotated and screened. The quality control index of sequencing data is the average sequencing depth of the target area, which is ≥100×, and the proportion of sites with the average depth of the target area >20×, which is >95%. The sequencing fragment was compared with the human reference genome hg19 by BWA software to remove the duplication. The GATK software was used for SNV, Indel, and genotype monitoring.

### 3.5 Sanger sequencing verification

About 4 mL venous blood sample was taken from the other members of the family and sent to BGI for sequencing. Sanger sequencing was used to verify the mutation results of proband and her family members. For the c.1072T > C variation of SLC6A20 gene, primer3 input was used to design primers in the upstream and downstream of the segment. PCR amplification was carried out and Sanger sequencing was performed on the products. The results were compared with the SLC6A20 gene standard sequence NM_020208.3 to verify the results of gene chip capture and high-throughput sequencing.

## 4 Results

### 4.1 Laboratory testing results

The laboratory testing results are shown in Table 1. For the two sisters, the results of anti-SSA antibody, anti-SSB antibody, Schirmer test, and Corneal staining were all positive. No red blood cells were found in the urine under the microscope. The concentrations of serum creatinine (Scr) and 24 h urine total protein were found to be elevated in both of them. For the two sisters the contents of oxalate, potassium ion, and calcium ions in the urine were normal. The urine sodium and chlorine concentrations for the two sisters were reduced.

**Table 1:** The laboratory testing results of proband and her sister

	Proband	Proband’s sister
pH value of morning urine	6.0	6.0
Urine protein	Positive	Negative
Urine red blood cells (per hpf)	0	0
Scr (µmol/L)	145	127
Serum uric acid (µmol/L)	315	352
24 h Urine total protein (mg)	1044.47	414.41
24 h Urinary calcium (mmol/24 h)	3.35	2.43
24 h Urinary calcium sodium (mmol/24 h)	97.02	105.29
24 h Urinary chlorine (mmol/24 h)	76.23	89.61
24 h Urinary oxalate (µmol/24 h)	2366.59	2532.7
24 h Urinary potassium ion (mmol/24 h)	35.69	31.82
24 h Urinary calcium ion (mmol/24 h)	2.43	3.35
Urine osmotic pressure (mOsm/kg)	146.3	154.4
Anti-SSA antibody	Positive	Positive
Anti-SSB antibody	Positive	Positive
Schirmer test	Positive	Positive
Corneal staining	Positive	Positive

### 4.2 Imaging examination

**Figure 1:**
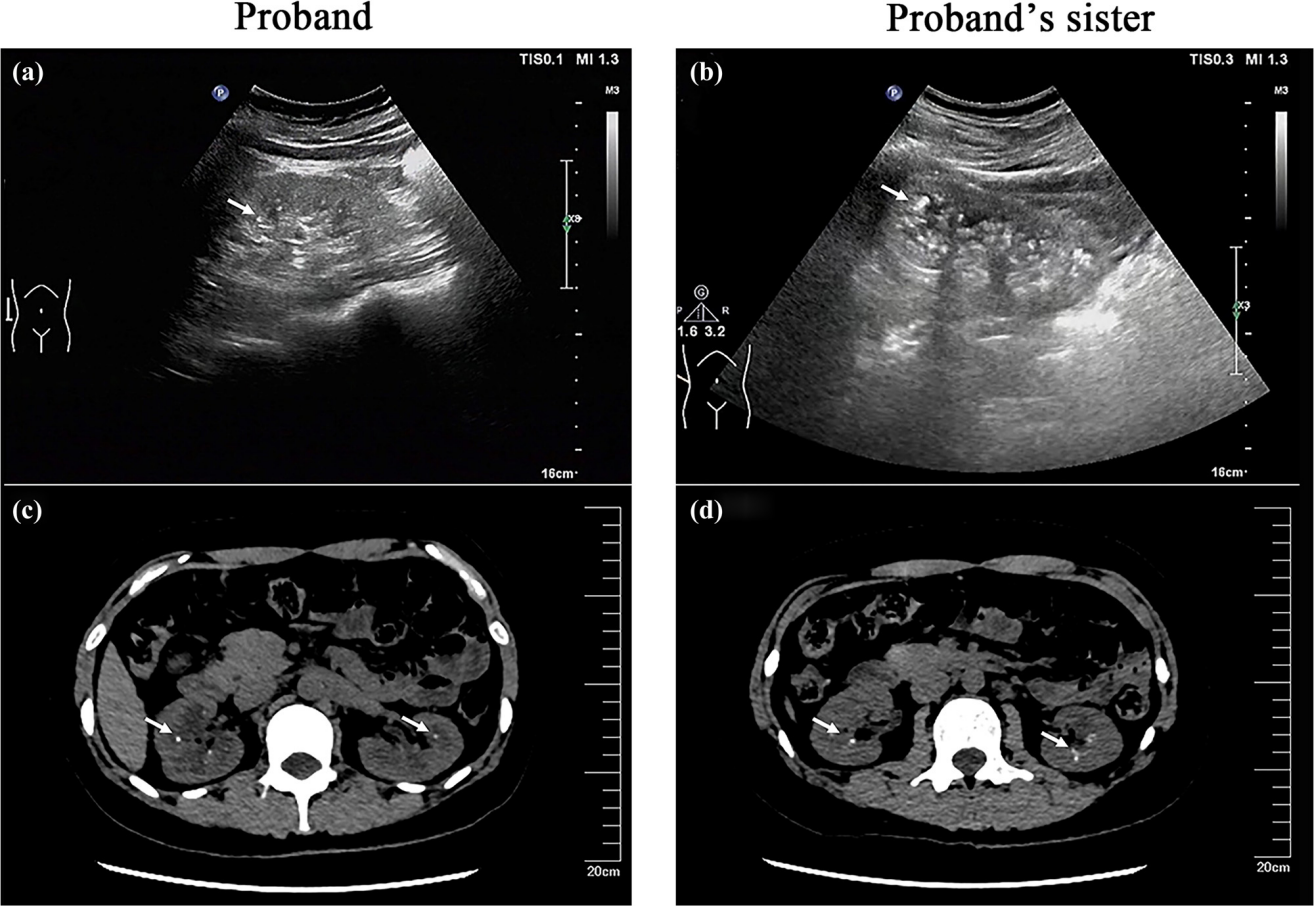
Imaging examinations of the kidneys for the proband and her sister. (a and b) Color Doppler ultrasonography of the kidney of the patient and his sister respectively; (c and d) 16 row spiral Computed Tomography scan images of the patient and her sister respectively. Multiple small stones were found in both kidneys. The arrow designates the stones.

The color Doppler ultrasound examination of the patient showed the shape of both the kidneys to be normal. The size of right kidney is 100 mm × 47 mm with a 12 mm thickness of renal cortex and the size of left kidney is about 101 mm × 47 mm with a 12 mm thickness of renal cortex. The echo of both renal parenchyma is increased. No obvious separation is found in both kidney collection systems. Multiple hyperechoic masses can be seen in both kidneys, the larger one is about 3 mm (Figure 1a). Computed tomography scan shows that the density of bilateral renal parenchyma is not uniform. Multiple spot or nodular high-density shadows are seen in renal parenchyma and renal calyces, and multiple kidney stones were considered (Figure 1c). The color Doppler ultrasound examination of the younger sister has found the size of the right kidney to be 106 mm × 48 mm with a 13 mm thickness of renal cortex and the size of the left kidney to be 95 mm × 53 mm with a 15 mm thickness of renal cortex. The echo of the parenchyma of both the kidneys is also increased. Two strong echo clusters can be seen in both the kidneys, and the maximum diameter of the larger one is about 5 mm (Figure 1b). Computed tomography scan shows that the size and shape of both kidneys were normal, and two high-density spots could be seen in renal parenchyma and calyces. Small stones in both kidneys were considered (Figure 1d).

### 4.3 Labial gland and renal pathology

**Figure 2:**
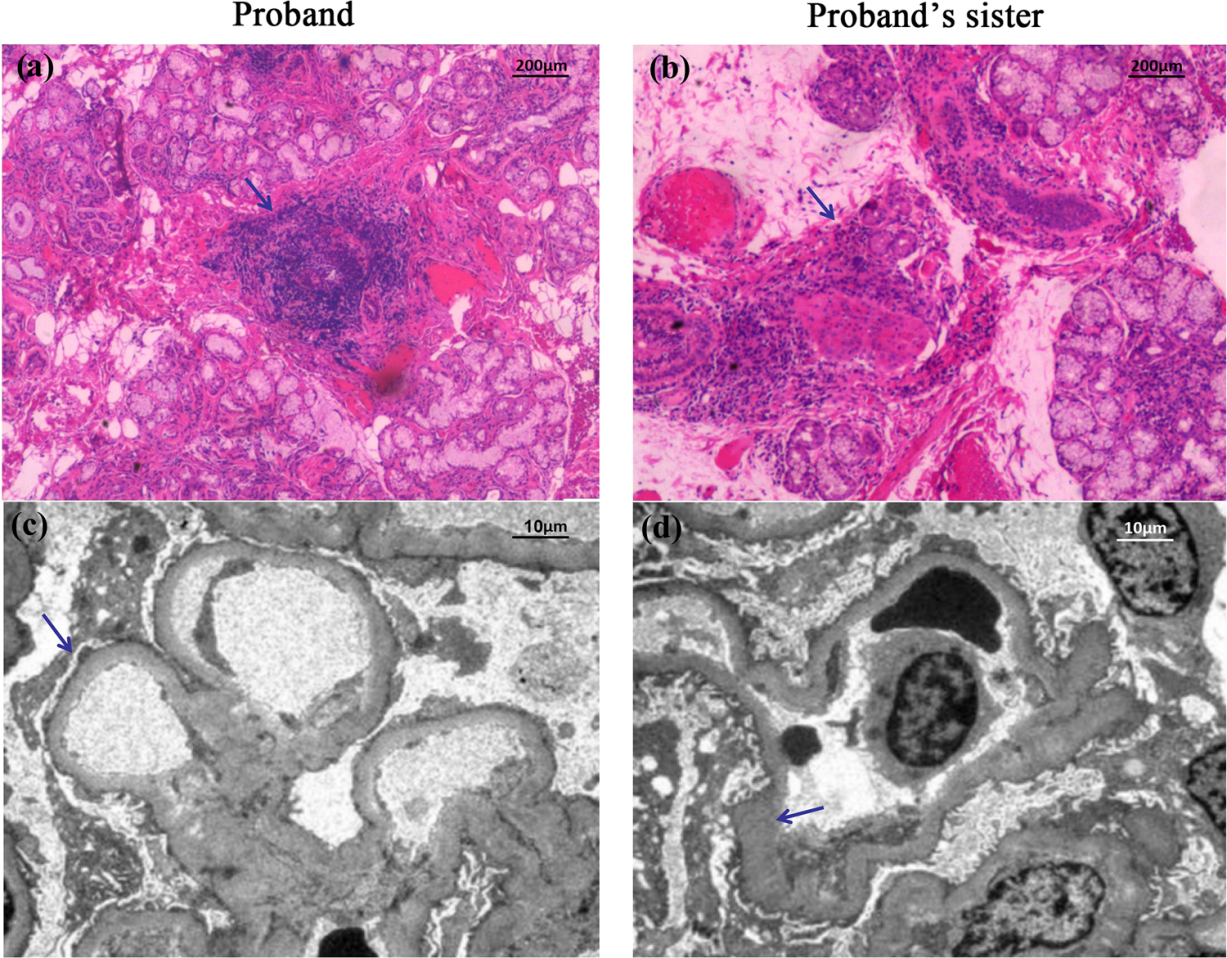
The pathological examination of labial gland and normal transmission electron microscope examination of kidney. (a and b) HE staining (×100) of the biopsy tissue of the patient and her sister's labial gland respectively. There are 50 lymphocytic foci in 4mm^2^, which are called as 1 foci. Under the microscope, there are 1 lymphocytic foci, so the histological examination of labial gland biopsy is positive; (c and d) The result of normal transmission electron microscope examination of the sisters' kidney tissue was similar. Diffuse homogeneous thickening of glomerular basement membrane and foot process fusion were seen, but there was no electron dense deposition (×2000). The arrow designates the lesions.

**Figure 3:**
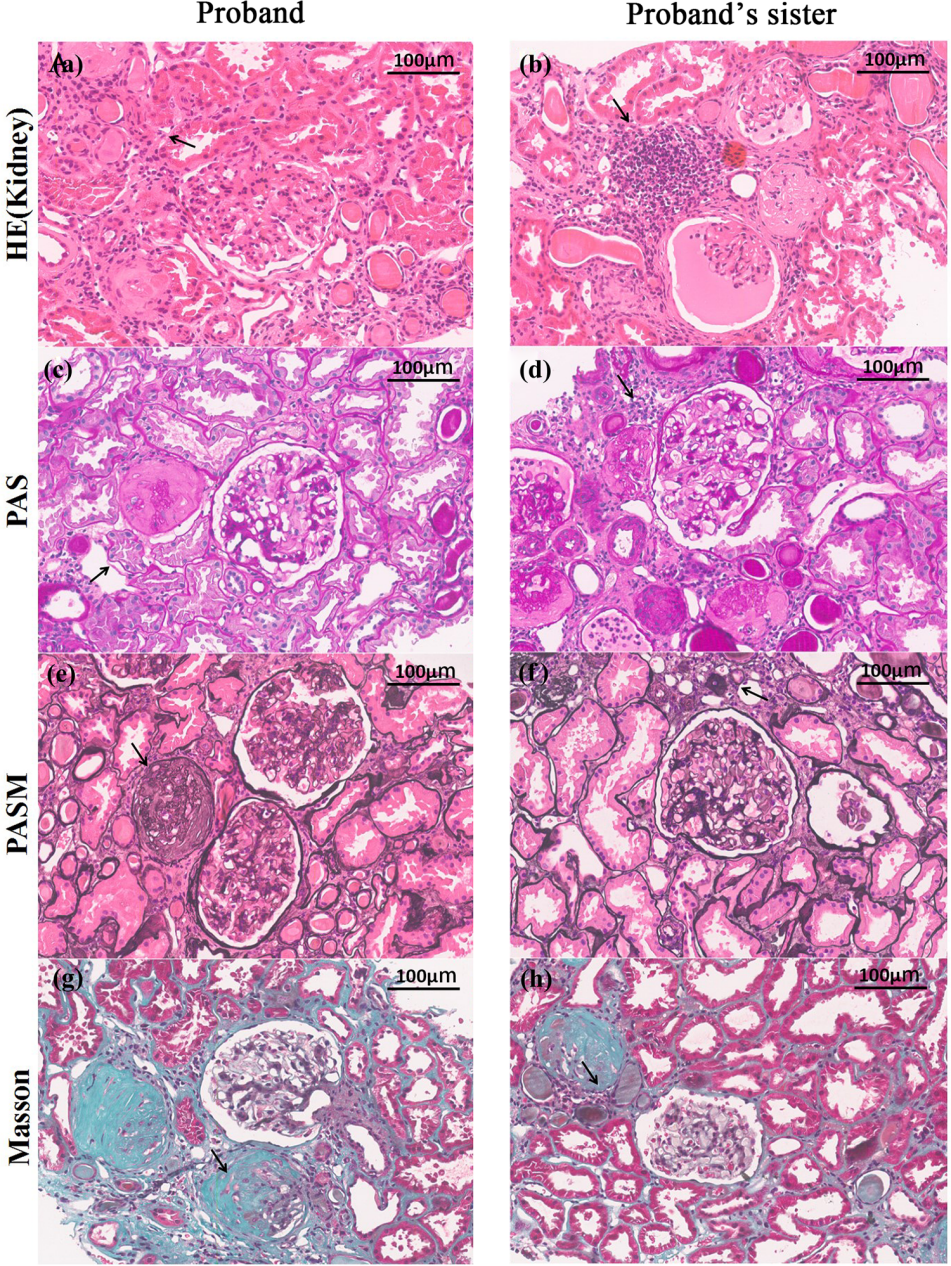
The pathological examination of kidney tissue. (a–h) HE, PAS, PASM, and Masson's staining (×200) of pathological sections of kidney tissue from the patient (a, c, e, g) and her sister (b, d, f, h) respectively. Under the microscope, interstitial lesions were mainly seen including moderate tubular atrophy and interstitial fibrosis. Glomerulopathy was slight and some glomerulosclerosis was seen. The arrow designates thelesions.

The pathological results of the labial gland and kidney biopsy of the patient were similar to those of her sister. The pathological results of the labial gland were as follows: (the lower labial gland) the structure of the labial gland existed under the microscope and the duct in the lobule slightly expanded. Many lymphocyte infiltrations were seen in the lobule. According to Chisholm standard, the pathological changes of the labial gland accorded with 4 degrees (Figure 2a and b). The renal pathological results showed that the glomerulus had a slight pathological change, with some glomerulosclerosis and moderate renal tubulointerstitial lesions (Figure 3). The normal transmission electron microscopy of the renal tissue of the patient indicated that the glomerular basement membrane was diffusely and homogeneously thickened. The foot processes were widely fused, and no electron dense deposition was found (Figure 2c). The result of the younger sister suggests that the glomerular basement membrane is diffuse with homogeneous thickening. Most of the foot processes are fused, and no electron dense deposition is found (Figure 2d).

### 4.4 Amino acid analysis results

Urine samples were obtained from ten healthy volunteers as health control for further analysis. Twenty kinds of amino acids in urine and blood sample were detected including glycine, proline, hydroxyproline, etc. There was no significant abnormality in the detection except hyperglyciuria in the proband and her mother (Table 2). We also investigated 132 kinds of organic acids in urine, including acetylglycine, propionylglycine, isoglutarylglycine, malonic acid, oxalic acid, glyceric acid, etc. (data are not shown here). All the organic acid contents in urine were normal in the detection and no hyperaciduria and glycerinuria were found.

**Table 2:** The detection of 20 kinds of amino acids in urine

Amino acid (µmol/L)	Ⅰ_1_	Ⅰ_2_	Ⅱ_2_	Ⅱ_3_	Ⅲ_1_	Ⅲ_2_	Health control (*n* = 10)
Alanine	166.1	407.6	61.1	43.6	83.6	102.7	425.1 ± 90.5
Argnine	14.5	34.8	5.9	2.2	23.9	6.4	79.2 ± 51.6
Aspartic acid	20.6	40.6	9.8	2.9	55.2	37.3	87.1 ± 35.1
Cysteine	27.8	15.7	5.0	7.8	31.5	67.2	16.1 ± 3.0
Glutamic acid	9.2	19.2	2.3	2.3	7.1	24.0	90.0 ± 20.0
Glycine	884.0*	9.0	98.3*	60.1*	9.3	4.1	11.9 ± 14.0
Histidine	410.6	553.4	38.9	52.3	18.3	91.7	294.6 ± 160.6
Homocysteine	0.1	0.3	0.0	0.0	0.2	0.1	3.2 ± 1.1
Hydroxyproline	0.2	65.9	2.2	10.0	21.7	52.4	52.7 ± 21.6
Isoleucine	7.3	23.0	1.2	1.6	6.6	3.2	8.8 ± 3.5
Leucine	20.6	65.0	3.2	3.9	88.3	121.9	71.8 ± 34.9
Lysine	61.9	415.3	5.2	14.3	214.0	76.3	445.4 ± 217.8
Methionine	4.9	11.3	0.8	0.8	3.1	5.6	6.8 ± 4.9
Phenylalanine	40.1	83.7	5.5	9.2	33.2	91.5	73.5 ± 52.9
Proline	3.3	25.2	1.6	4.1	17.3	9.8	39.8 ± 10.8
Serine	185.2	277.9	21.3	22.2	223.5	317.6	449.6 ± 130.6
Threonine	109.1	188.8	11.8	24.6	5.1	11.4	113.5 ± 56.7
Tryptophane	40.8	65.2	5.8	8.0	0.6	10.8	83.5 ± 22.4
Tyrosine	73.4	93.6	6.9	15.0	33.7	110.8	120.0 ± 60.0
Valine	30.2	76.9	0.8	3.4	7.3	25.7	50.4 ± 17.0

The amino acid concentrations were investigated by GC–MS. ^*^Abnormal level.

### 4.5 Sequencing results of all exons

**Figure 4:**
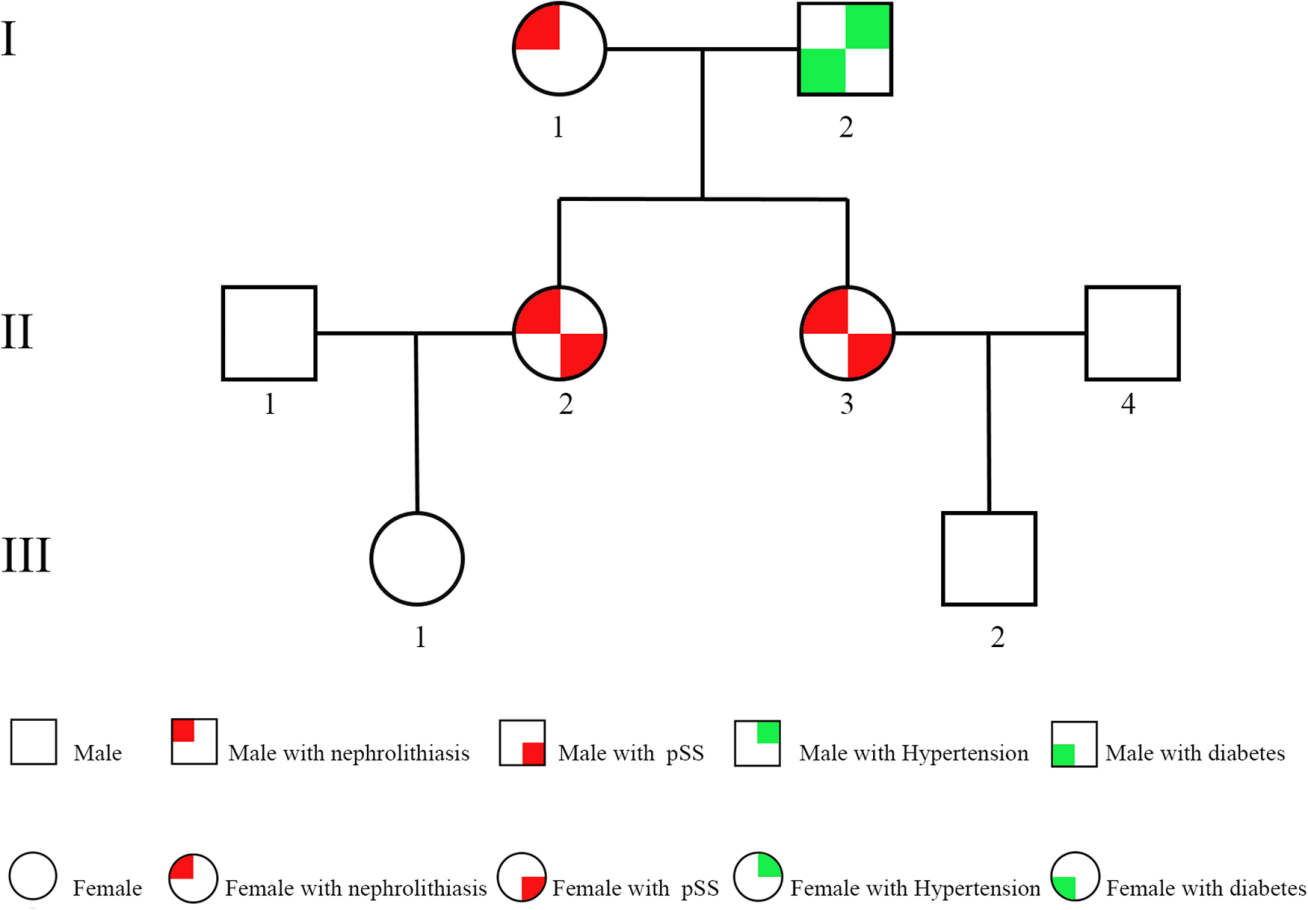
Family diagram. The patient (2) and her sister (3) were characterized by nephrolithiasis and primary Sjogren's syndrome while the mother (1) showed only the phenotype of nephrolithiasis. The rest members of the family are healthy.

National Center for Biotechnology Information database of Single Nucleotide Polymorphism, Haplotype Map of the human genome, the NHLBI GO Exome Sequencing Project, the Exome Aggregation Consortium, 1,000 human genome database, and database of 100 Chinese healthy adults were used to filter the data and found that the proband (Ⅱ_1_) and her sister (Ⅱ_2_) in the family had a same heterozygous variation of c.1072T > C (p.Cys358Arg) in the NM_020208.3 sequence of SLC6A20 gene exon (Figure 4). InterVar is a bioinformatics software tool for clinical interpretation of genetic variants by the American College of Medical Genetics and Genomics and the Association for Molecular Pathology 2015 guideline (http://wintervar.wglab.org/) [26]. The SLC6A20 c.1072T > C (p.Cys358Arg) is predicted with “Uncertain significance” and we did not find pathogenic variation reported in literature.

### 4.6 Sanger sequencing verification results

**Figure 5:**
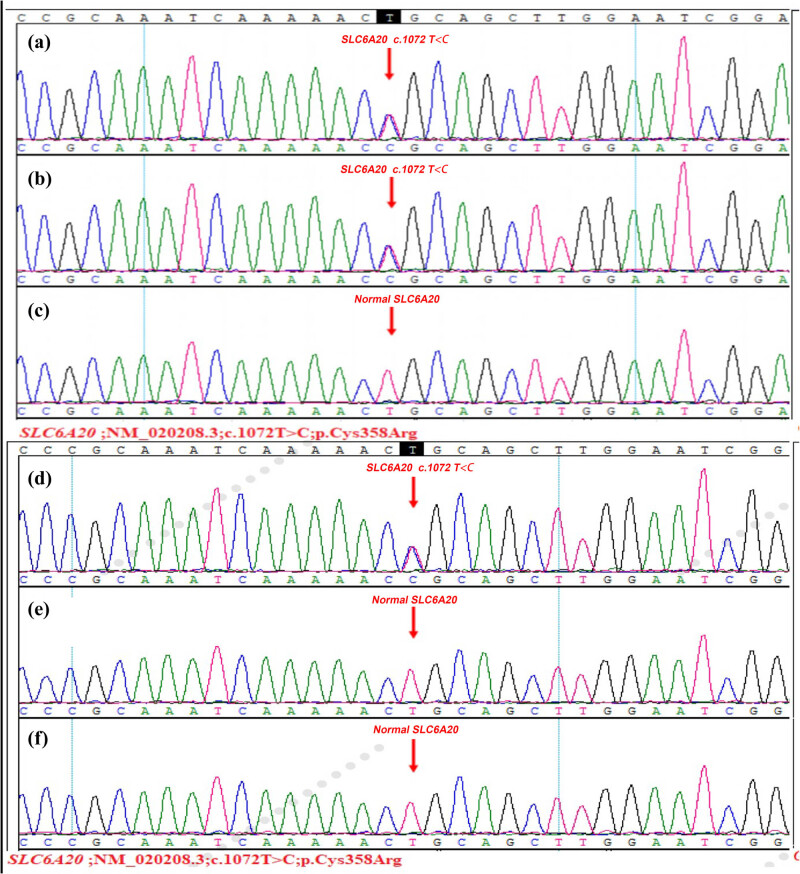
DNA sequencing and Sanger sequencing verification. (a, d) The c.1072T > C heterozygous variation in the SLC6A20 gene exon from the proband (2) and her sister (3). (b) The c.1072T > C heterozygous variation in the proband's mother (1). (c, e, f) The normal gene in the proband s father (2), son and nephew.

The results showed that there was a heterozygous variation of c.1072T > C(p.Cys358Arg)in the NM_020208.3 sequence of SLC6A20 gene exon of the proband’s mother (Ⅰ_1_) and the rest of the relatives did not detect the gene variation (Figure 5).

## 5 Therapeutic intervention and follow up

The sisters received 20 mg of prednisone once per day, 20 mg of olmesartan once per day, and 2.0 g of potassium citrate granules three times a day for 3 months. The level of their serum creatine, urine protein, and the condition of nephrolithiasis was maintained stable till June 2021.

## 6 Discussion

SLC6A20 gene in human is located on chromosome 3p21.3, which is a member of SLC6 family of membrane transporters and the product of this gene expression is transporter protein of sub-amino acid transporter system [21]. SLC6A20 is mainly expressed in intestine and kidney, which plays an important role in the uptake or reabsorption of proline and hydroxyproline. It is a sodium chloride-dependent transporter that mediates the co-transport of l-proline with two sodium ions and one chloride ion. SLC6A20 transports hydroxy l-proline and betaine with relatively high apparent affinity, but does not transport glycine [27]. In view of its important role in proline transport, SLC6A20 is associated with some forms of autosomal recessive sub-amino aciduria [22]. For example, the iminoglycinuria is reported to be an autosomal recessive abnormality of renal transport of glycine, proline, and hydroxyproline [28]. Meanwhile, the previous study has demonstrated four different transporters for proline and glycine in the kidney, including the common transporter SLC36A2, the specific proline transporter SLC6A20, the specific glycine transporter SLC6A18, and the general neutral amino acid transporter SLC6A19 [29]. Their study established a model to predict a total capacity of 100% for proline uptake of human kidney, comprising SLC36A2 (43%), SLC6A20 (28.5%), and SLC6A19 (28.5%). The optimized model suggests that SLC6A20 has a weaker capacity for proline uptake than SLC36A2. Thus, the mutation of SLC36A2 is the main determinant factors of the hyperglycinuria and iminoglycinuria phenotypes. So, inactivating mutations of SLC6A20 might account for iminoglycinuria or the degree of urinary solute loss. Actually, main variation locations of SLC6A20 have been reported in ClinVar such as NM_020208.3(c.*3436C > T) and NM_020208.3(c.354 + 2T > A) which may cause hyperglycinuria or iminoglycinuria.

In this study, urine analysis was performed on this family. We found that the proband and her mother had hyperglycinuria without hyperprolinuria and hyperhydroxyprolinuria. This is consistent with previous studies that have reported that iminoglycinuria and hyperglycinuria are associated with nephrolithiasis, hypertension, glycosuria [22]. Moreover, glycine excretion values exceeding the normal level is corresponding with tubular re-absorption rates below 92.9%. The free glycine might increase the urinary oxalate concentration and promote an oxalate calculus formation [29]. Meanwhile, we only found that the glycinuria level of the mother was much higher than that of the proband which might explain why the kidney stones of the proband and her mother were more serious, while the kidney stones of the sister was less and smaller. Therefore, we hypothesized that the hyperglycinuria caused by SLC6A20 gene mutation is responsible for the kidney stone of this family. No family members had hyperprolinuria and hyperhydroxyprolinuria and the proband’s sister even had no hyperglycinuria. This phenomenon might be due to the heterozygosity of SLC6A20 gene variants. Severe hypoglycineuria will only occur when there are two defective alleles of the co-transporter of glycine, proline, and hydroxyproline. We also noticed that the urine composition analysis of the sisters showed that the contents of oxalate, potassium ion, and calcium ions in the urine were normal (Table 1). The urine sodium and chlorine concentrations for the two sisters were reduced. The data indicated that the excretion of sodium and chloride ions was reduced, which may be related to the abnormal transport of the substances in coordination with sodium and chloride ion transport in the kidney. In addition, the excretion of citric acid in the urine of the two sisters was in the range of 27.6–56.8 mg/24 h. As the urinary citrate excretion less than 320 mg/24 h is considered as hypocitruria, the hypocitruria of the case might participate in the formation of nephrolithiasis. However, the patients did not excrete any kidney stone and the composition of it could not be verified.

Interestingly, pSS was confirmed by pathological biopsy in both the proband and her sister in this family study. Renal involvement in Sjogren’s syndrome is mainly manifested by distal tubular acidosis, which results in the decrease of hydrogen ion secreted by the distal renal tubule, resulting in the compensatory increase of potassium ion in urine and the excretion of calcium ion, which leads to periodic hypokalemic paralysis. Yet, the sisters did not have these manifestation and the pH value of their morning urine were both 6.0 (Table 1). Therefore, we concluded that the nephrolithiasis of this family had less relationship with impairment of the acidification function of distal renal tubular acidosis caused by pSS.

## 7 Conclusion

In brief, each population has a mutation library. Most of the individual mutations (i.e., polymorphism) will have limited impact, but the combination with other alleles may promote or avoid body abnormalities. The heterozygous variation of c.1072T > C in SLC6A20 gene is one of the causes of kidney stone, and it may also cause the abnormal transport of amino acids or sub-amino acids in the kidney. In conclusion, the nephrolithiasis associated with SLC6A20 mutation may due to glycinuria or other pathways. However, the exact genetic mechanism of SLC6A20 gene and kidney stone remains to be further explored. We plan to generate the SLC6A20 c.1072T > C knock-in mice and introduce the nephrolithiasis model in our further study to validate.
